# Effects of Acute Supramaximal Cycle Exercise on Plasma FFA Concentration in Obese Adolescent Boys

**DOI:** 10.1371/journal.pone.0129654

**Published:** 2015-06-15

**Authors:** Georges Jabbour, Horia-Daniel Iancu, Anne Paulin, Jean-Marc Lavoie, Sophie Lemoine-Morel, Hassane Zouhal

**Affiliations:** 1 School of Kinesiology and Leisure, Faculty of Health Sciences and Community Services, Université de Moncton, Moncton, New-Brunswick, Canada; 2 Department of Kinesiology, University of Montreal, Montreal, QC, H3C 3J7, Canada; 3 Movement Sport and Health Sciences Laboratory, University of Rennes 2-ENS Cachan, Rennes, France; INIA, SPAIN

## Abstract

**Aims:**

The aims of the present study are 1) to evaluate the free fatty acid (FFA) profile and 2) to determine the relative anaerobic and aerobic contributions to total energy consumption during repeated supramaximal cycling bouts (SCE) in adolescent boys with different body weight statuses.

**Materials and Methods:**

Normal-weight (NW), overweight (OW), and obese (OB) adolescent boys (n =15 per group) completed a SCE sessions consisted of 6 x 6s maximal sprints with 2 min of passive rest between each repetition. Plasma FFA levels were determined at rest, immediately after a 10 min warm-up, and immediately at the end of SCE. The anaerobic and aerobic contributions (%) were measured via repeated SCE bouts. Insulin resistance was calculated using the homoeostatic model assessment (HOMA-IR) index.

**Results:**

The FFA concentrations measured immediately after SCE were higher in the OB group than in the OW and NW (p<0.01 and p<0.01, respectively) groups. Moreover, the anaerobic contributions to SCE were significantly lower in obese adolescents (p<0.01) and decreased significantly during the 2^nd^, 3^rd^ and 4^th^ repetitions. The FFA levels were significantly associated with the HOMA-IR index and aerobic contribution among adolescent boys (r=0.83 and r=0.91, respectively, p<0.01).

**Conclusion:**

In contrast to the NW and OW groups, there is an increase in lipid mobilization and sift to aerobic energy metabolism during SCE in the OB group.

## Introduction

High-intensity exercise such as supramaximal exercise is gaining popularity in the context of obesity management. Based on data provided by post intervention metabolic measures, this model of exercise appears to be a valuable approach for managing obesity. Indeed, Whyte et al. [1] demonstrated that very high-intensity sprint interval training improved several metabolic risk factors such as an increase in resting fat oxidation rate in the fasted state and a decrease in resting carbohydrate oxidation in the fasted state compared with baseline. This high-intensity training also decreased waist and hip circumferences in overweight and obese sedentary men and illustrated the potential of this alternative exercise model to improve lipid utilization in individuals with excess body weight. However, no information describing true metabolic adaptations (e.g. fat and carbohydrate oxidation), during exercise is available.

Previous studies reported that in response to supramaximal exercise in normal-weight individuals, a progressive decline in plasma free fatty acid (FFA) was accompanied by increased blood glucose turnover responsible for energy yield [2]. In fact, supramaximal exercise is a brief event, and in order to meet the high ATP demand (*e*.*g*., adenosine diphosphate [ADP]), phosphorylation from PCr hydrolysis and glycolysis is necessary [3]. This is reflected by high anaerobic contributions to total energy expenditure [4]. In the context of obesity, several factors such as reduced motor-unit activation [5], premature fatigue and reduced activation of type II muscle fibers [6] may affect the metabolism profile. Indeed, anaerobic capacity, which is the determinant factor of performance during such exercise, is affected by obesity [7]; high-intensity training enhances anaerobic capacity in overweight and obese men [1]. The mechanism(s) underlying these adaptations are likely the result of skeletal muscle adaptations involving marked increases in skeletal muscle capacity for glycolytic enzyme content [8], increases in anaerobic fitness, and increases insulin sensitivity [9].

Given that glycolytic activity may be altered by obesity, it seems interesting to address lipids, as an alternative energy nutrient to working muscles during supramaximal exercise. Lipid utilization is modulated by the availability of FFA in circulating blood. In lean healthy humans greater hydrolysis of adipose tissue triglycerides to FFA suggests that the availability of circulating FFA can potentially meet the oxidative needs of working muscles during submaximal exercise [10]. Additionally, simultaneous evaluation of the maximal activation of both energetic systems (e.g., anaerobic vs. aerobic) during supramaximal exercise may allow us to better understand anaerobic/aerobic interactions and provide information regarding the metabolic adaptation in obese individuals during such exercise.

As such, the present work aims to evaluate plasma FFA as well as anaerobic and aerobic profiles in response to supramaximal cycling exercise in overweight, obese and normal-weight adolescent boys.

## Materials and Methods

Forty-five healthy adolescent boys were recruited from several high schools in Lebanon. To prevent any maturation variability between them, we chose children in the age range of 13–14 years, who were all at the same Tanner stage (Stage 3) [11]. Written informed consent was obtained from the parents of each subject before the study, and the survey was approved by the Ethical Committee on Human Research (*ECHR*) of the University of Balamand (Lebanon). The inclusion criteria for participation were sedentary (participating in <1 h per week of structured exercise, as assessed by the International Physical Activity Questionnaire [12], no metabolic, cardiovascular or chronic health problems, no drug consumption before the study, and no smoking (information obtained from their family doctor). Before entering the protocol, the adolescents were familiarized with all testing equipment and procedures. The exercise testing was conducted on 4 different days (D1, D2, D3 and D4), each separated by a period of 8 days. Subjects were asked to rest the day before all testing and to be well hydrated.

### Anthropometric measurements

Body mass was measured to the nearest 0.1 kg with the subject in light clothing without shoes using an electronic scale (Kern, MFB 150K100). Height was determined to the nearest 0.5 cm with a measuring tape fixed to the wall. The body mass index (BMI) was calculated as the ratio of mass (kg) to height^2^ (m^2^). The percent of body fat was estimated from 3 skinfold thicknesses (biceps, triceps and sub-scapular) as previously described [13]. After the determination of body composition, the participants were separated into three groups based on the criteria previously described [14]: the normal-weight (NW) (n = 15; body fat % < 22) group, the overweight (OW) (n = 15; body fat % = 22–25) group and the obese group (OB) (n = 15; body fat % >26). The fat free mass was calculated by subtracting the fat mass from the body mass.

### Maximal Test

On D1 subjects performed a maximal test on an upright cycle ergometer (Monark ergomedic 839E electronic test cycle, Sweden) to determine their maximal oxygen consumption (VO_2max_) and to ensure that groups were matched for fitness level. The initial power was set at 60 Watts and progressively increased by 20 Watts every 2 min until exhaustion with a maintained pedaling rate of 60 revolutions/min (rpm). A breath-by-breath automated metabolic system (CPX, Medical Graphics, St. Paul, Minnesota, USA) was used to determine the VO_2max_. Achievement of VO_2max_ was accepted when subjects fulfilled at least three of the following criteria: a plateau in VO_2_ despite an increase in exercise intensity, a respiratory exchange ratio greater than 1.1, a maximal HR above 90% of the predicted maximal theoretical HR (220-age in years), a blood lactate concentration higher than 8.0 mmol·L^-1^ and the apparent exhaustion of the subject [15].

### Submaximal test

On D2, we measured steady state VO_2 uptake_ at a constant submaximal power (below the VO_2max_) using a breath-by-breath automated metabolic system (CPX, Medical Graphics, St. Paul, Minnesota, USA). After a 3-min resting baseline period, subjects started pedaling at the established power, which was maintained for 10 min at a pedaling rate of 60 revolutions/min (rpm). The 10-minute exercise bouts were completed 6 times for each subject at powers of 30%, 40%, 50%, 60%, 70% and 80% of their individual VO_2max_ power and were separated by 5 minutes resting period, and the measurements for each subject were plotted separately and visually checked for linearity as described elsewhere [16]. This demonstrates that our measured VO_2 uptake_ for a 10-min exercise was a steady-state value equaling VO_2 demand_ (total rate of energy release). We therefore conclude that VO_2 demand_ increased linearly with power for all subjects in the examined range. This analysis allowed for the calculation of the accumulated oxygen deficit (AOD) (measured in ml O_2_ equivalents per kg) for each cycling interval by calculating the difference between VO_2 demand_ for the respective power (from extrapolation of the calculated relationship) and measured O2 cost. The linear relationship between steady state VO_2_ values and cycling power was extrapolated and used to estimate energy demand during supramaximal cycling exercise (SCE).

### Force-Velocity Test (F/V)

On D3, and before the beginning of the test, adolescents warmed-up for 10 min at a submaximal power between 55–65 Watts. The F/V test was performed on a cycle ergometer using a technique adapted from a previous study [17]. This test consists of a succession of supramaximal bouts of approximately 6 seconds in duration with a starting load of 2 kg and with the exercise loads increasing by 2 kg after each bout until exhaustion. A passive recovery (5 min) was allowed between successive bouts. The velocity for each bout was recorded every two seconds. Only the highest velocity (V) was recorded for each load using an electronic counter (MEV 2000). Power output was calculated by multiplying the load and speed, and a power curve was then compiled for each bout. The optimal load (F) corresponding to the maximal power was used for the SCE performed on D4.

### Supramaximal cycling exercise (SCE)

On D4, each subject performed a repeat sprint cycling test (6-repetitions of 6-second maximal sprints with 2 min of passive rest between each repetition). Each SCE bout lasted six seconds, and participants were asked to pedal at maximal velocity against the load determined during D3. The SCE began with a 5 minute warm-up of continuous cycling at moderate intensity (40% of their individual VO_2max_ power) followed by 2 minutes of recovery. The repeat sprint cycling test was conducted under the supervision of a member of the research team, and velocities (in RPM) were recorded for each second of the bout in order to ensure that said velocities were constant. This form of very short supramaximal sprint exercise was tolerated by all of our participants and may be more relevant to their physical activity behaviors. During SCE, the mean of all power (PO_mean_) exerted by the participants was calculated.

The experimentation started at approximately 9 am, 3 hours after a standardized breakfast (10 kcal/kg, 55% carbohydrate, 33% lipid and 12% protein). In the present study we controlled whether all participants ate a breakfast. Venous blood samples from an antecubital vein were drawn at rest, and then, the adolescents warmed-up for 10 min at a submaximal power between 55–65 watts (W) (corresponding to ~ 60% of VO_2max_). Venous blood samples were drawn again immediately after the warm-up period and immediately after the end of the test. Subsequently, the repeated cycling sprint test was performed, and the subjects were asked to cycle as fast as possible with 2 min of passive rest between each repetition.

### Blood analyses

At each extraction, the blood was collected in a vacutainer tube containing Ethylene Diamine Tetra Acetic Acid *(EDTA)*. Hematocrit (Ht) was measured three times for each blood sample by micro-centrifugation *(JOUAN-HEMAC)*. Plasma from the venous blood samples was separated by centrifugation at 3000 g for 20 min (4°C) (*ORTO ALRESA mod*. *Digicen*.*R*, *Spain*). Aliquots were immediately frozen and stored at -80°C for use in subsequent chemical analyses. The blood lactate concentrations obtained at rest, after the warm-up and at the end of SCE were determined enzymatically using a lactate analyzer *(Microzym*, *Cetrix*, *France)*. Rest plasma insulin concentrations were measured in the centralized laboratory by a radio-immunoassay procedure (Phaadebas Insulin Kit; Pharmacia Diag-nostics AB, Piscataway, NJ). Plasma FFA was determined using a kit from Roche Diagnostics (Mannheim, Germany) by enzymatic colorimetric assay according to the manufacturer’s instructions. The rest plasma glucose was assayed by the glucose oxidase method (Boehringer kit, Meylan, France). Plasma lactate and FFA values were corrected for plasma volume changes using the equation of Van Beaumont [18]. An estimate of insulin resistance was calculated by the homoeostatic model assessment (HOMA-IR) index as: [insulin (μU/mL) × glucose (mmol·L^-1^)]/22.5. To distinguish normal from impaired insulin sensitivity, a HOMA-IR greater than 2.5 and greater than 4.0 were the cut-off points used in children and adolescents, respectively [19].

### Calculation of relative energy expenditure

The VO_2 demand_ values of SCE were estimated individually by extrapolating the linear relationship between the power and the VO_2 demand_ values established during the constant submaximal power exercises. The accumulated VO_2 uptake_ and the accumulated VO_2 demand_ were taken as the VO_2 uptake_ and the VO_2 demand_ integrated over the entire supramaximal cycling exercise bout [16]. The accumulated oxygen deficit (AOD) (measured in ml O_2_ equivalents per kg) was equal to the accumulated VO_2 demand_ minus the accumulated VO_2 uptake_. This allowed for a measurement of anaerobic (AOD) and aerobic (VO_2_) energy contributions throughout the four SCE repetitions [20]. In this study, results are given only from four supramaximal cycling exercise bout given that some of participants felt discomfort by wearing mask destined for gas analysis. Then, the mask was removed to allow participants to complete the entire session bouts.

### Statistical analysis

Data were summarized as the mean and the standard deviation (SD). The data were analyzed using Statistica 7.1 software. After testing for the normal distribution (Kolmogorov—Smirnov test), differences within and between the NW, OW and OB groups were analyzed using a two-way analysis of variance for repeated measurements. After confirming significant group differences over time, the Newman-Keul’s post hoc test was performed. The power of the correlation analyses was calculated using a Pearson test (for parametric data). A value of p<0.01 was accepted as the minimal level of statistical significance.

## Results

Subject characteristics are displayed in Table 1. Age and height were similar among subjects in the three groups. Mean power and VO_2max_, expressed per unit of fat free mass, did not significantly differ among the three groups (Table 1).

**Table 1:** Physiological characteristics.

	Normal-weight(n = 15)	Overweight (n = 15)	Obese (n = 15)	Group effect (df = 2)	
**Anthropometric characteristics**				F	P
Age (years)	13.6(0.1)	13.4(0.1)	13.6(0.3)	1.7	0.31
Height (cm)	162.9(6.2)	164.4(10.4)	168.9(9.6)	1.6	0.33
Body mass (kg)	50.5(5.2)	67.0(10.0)^a^	88.7(14.7)^a b^	11.2	<0.0001
BMI (kg.m^-2^)	18.9(1.1)	24.5(1.5)^a^	30.8(2.3)^a b^	19.1	<0.0001
FM (%)	16.0(1.9)	24.5(1.6)^a^	31.0(3.0)^a b^	23.9	<0.0001
FFM (kg)	43.0(6.0)	52.0(9.0)^a^	62.0(8.0)^a b^	21.4	<0.0001
**Variables obtained during maximal test**					
VO_2max_ (ml)	2247(120)	2572(220)^a^	2927(199)^a b^	11.8	<0.0001
V VO_2max_ (ml·min^-1^·kg^-1^)	44.5 (4.6)	38.4(5.3)^a^	33.0(5.0)^a^	19.8	<0.0001
VO_2max_ (ml·min^-1^.kg FFM^-1^)	52.1 (4.1)	48.7(5.7)	48.1(4.4)	1.6	0.14
Lactate (mmol·L^-1^)	8.4 (4.4)	8.9(3.4)	9.8(3.3)^a b^	21.32	<0.001
HRpeak (beats·min^-1^)	202.9 (4.1)	201.2(3.7)	201.3(4.1)	0.4	0.64
RERpeak	1.22 (0.05)	1.18(0.03)	1.20(0.04)	1.21	0.29
PO_mean_ (W)	473 (92)	524(137)^a^	649(89)^a b^	17.9	<0.0001
PO_mean_ (W·kg^-1^)	9.4 (1.6)	7.7(1.1)^a^	7.3(0.8)^a^	19.8	<0.001
PO_mean_ (W·kg^-1^·FFM^-1^)	11.1(1.3)	10.4(1.1)	10.8(1.4)	1.32	0.13

Values are mean (standard deviation). *BMI*: Body Mass Index, *FM*: Fat Mass, *FFM*: Fat Free Mass, *VO*_*2max*_: Maximal oxygen consumption, *HRpeak*: peak heart rate, *RER*: respiratory exchange ratio, *PO*_*mean*_: The mean of all powers developed during the SCE.

Significant difference with NW: (^a^: p<0.01). Significant difference with OW (^b^: p<0.01).

The plasma FFA levels at rest were significantly higher in the OB group than in the NW (p<0.01) and OW (p<0.01) groups (Table 2). However, immediately after the warm-up, the FFA values in the OB group were lower than in the NW and OW groups (p<0.01) and decreased compared to the resting levels (p<0.01). In the NW and OW groups the FFA concentrations after the warm-up were similar to the resting values. After the SCE, the FFA values increased from resting values only in the OB group (+ 0.06 mol·L^-1^, p<0.01). The exercise FFA levels was significantly higher in the OB group than in the OW (p<0.01) and NW (p<0.01) groups.

**Table 2:** Metabolic parameters of adolescent boys.

	Normal-weight (n = 15)	Overweight (n = 15)	Obese (n = 15)	Group effect (df = 2)	
				F	P
**Rest**					
Glucose (mmol·L^-1^)	4.3(0.1)	4.3(0.1)	4.8(0.1)^a b^	18.7	<0.0001
Insulin (μmol·mL^-1^)	12.7(3.2)	13.1(4.2)	22.7(0.3)^a b^	21.6	<0.0001
FFA (mol·L^-1^)	0.04(0.01)	0.05(0.01)^a^	0.07(0.01)^a b^	18.2	<0.0001
HOMA-IR	2.2(0.1)	2.3(0.1)	4.7(1.3)^a b^	21.7	<0.0001
Lactate (mmol·L^-1^)	1.3(0.3)	1.3(0.5)	1.3(0.7)	1.3	0.53
**Warm-up**					
FFA (mol·L^-1^)	0.05(0.01)	0.05(0.01)	0.03(0.01)^a b c^	19.7	<0.0001
Δ1FFA (mol·L^-1^)	0.01(0.01)	0.01(0.01)^a^	-0.03(0.01)^a b^	22.6	<0.0001
Lactate (mmol·L^-1^)	4.4(1.2)	4.5(1.1)	4.5(2.2)	1.2	0.1
**Supramaximal cycling exercise**					
FFA (mol·L^-1^)	0.03(0.07)	0.04(0.01)	0.09(0.01)^a b c^	16.7	<0.0001
Δ2FFA (mol·L^-1^)	-0.03(0.01)	-0.01(0.03)	0.06(0.09)^a b^	19.6	<0.0001
Lactate (mmol·L^-1^)	11.4(1.2)	11.6(2.3)	11.8(1.3)	3.2	0.5

Values are mean (standard deviation). *FFA*: Free fatty acid, *HOMA-IR*: homoeostatic model assessment index, *Δ1*: the change in analyses measured between warm-up and rest, *Δ2*: the change in analyses measured between supramaximal cycling exercise and warm-up.

Significant difference with NW (^a^: p<0.01). Significant difference with OW (^b^: p<0.01). Significant difference with rest (^c^: p<0.01).

Resting insulin and glucose concentrations were significantly higher in the OB group compared to the NW and the OW (p<0.01, respectively) groups (Table 2). In addition, the HOMA-IR (insulin resistance) was significantly higher in OB group compared to the OW and NW groups. For plasma lactate measures, the values obtained after the warm-up period, and the SCE did not differ statistically between groups.

For the OB group, VO_2 uptake_ increased significantly during the 2^nd^, 3^rd^ and 4^th^ repetitions compared with values obtained during the 1^st^ repetition SCE (p<0.01) (Table 3). In contrast, the AOD decreased for the OB group during the 2^nd^, 3^rd^ and 4^th^ repetitions (p<0.01). Following SCE, the NW and OW groups demonstrated significantly higher AOD values compared with the OB group (p<0.01, respectively). In addition, the VO_2 uptake_, the AOD, and the aerobic and the aerobic contributions determined during SCE remained constant during all repetition bouts for the NW and OW groups (Table 3).

**Table 3:** Variables obtained during the supramaximal cycling exercise.

	Normal-weight (n = 15)	Overweight (n = 15)	Obese (n = 15)	Group effect (df = 2)	
				F	P
**1^st^ repetition**					
HR (beats·min^-1^)	113.4 (4.1)	113.1(2.8)	112.1(2.8)	1.4	0.6
PO (W·kg^-1^·FFM^-1^)	11.1 (1.3)	11.4(1.1)	10.9(1.4)	22.7	0.3
VO_2 uptake_ (mL·min^-1^·kg^-1^)	6.1 (3.1)^a^	6.2(2.1)^a^	14.1(3.7)	19.2	<0.0001
AOD (mL·min^-1^)	104.5 (3.4)^a^	105.6(1.4)^a^	72.4(1.4)	23.7	<0.0001
Aerobic contribution (%)	6^a^	7^a^	17	19.7	<0.0001
Anaerobic contribution (%)	94^a^	93^a^	83	20.1	<0.0001
**2^nd^ repetition**					
HR (beats·min^-1^)	114.4(3.1)	113.2(3.1)	113.1(2.9)	1.3	0.4
PO (W·kg^-1^·FFM^-1^)	11.2(1.3)	11.2(1.1)	11.1(1.4)	20.7	0.3
VO_2 uptake_ (mL·min^-1^·kg^-1^)	8.1(3.1)^a^	8.3(1.1)^a^	16.1(3.7)^b^	20.2	<0.0001
AOD (mL·min^-1^)	103.1(0.5) ^a^	105.1(0.3) ^a^	70.4(0.5)^b^	22.7	<0.0001
Aerobic contribution (%)	7^a^	8^a^	19^b^	18.7	<0.0001
Anaerobic contribution (%)	93^a^	92^a^	81^b^	20.4	<0.0001
**3^rd^ repetition**					
HR (beats·min^-1^)	116.4(4.1)	113.1(1.1)	117.9(1.8)	1.8	0.6
PO (W·kg^-1^·FFM^-1^)	11.4(2.1)	11.2(1.1)	11.6(2.1)	22.3	0.6
VO_2 uptake_ (mL·min^-1^·kg^-1^)	7.1(3.1)^a^	7.3(1.1)^a^	19.1(3.7)^b^	19.2	<0.0001
AOD (mL·min^-1^)	103.9(5.3)^a^	105.6(0.9)^a^	67.4(1.2)^b^	20.9	<0.0001
Aerobic contribution (%)	7^a^	8^a^	22.6^b^	18.9	<0.0001
Anaerobic contribution (%)	93^a^	92^a^	77.4^b^	20.9	<0.0001
**4^th^ repetition**					
HR (beats·min^-1^)	117.4(3.1)	118.4(2.1)	118.9(0.8)	1.9	0.6
PO (W·kg^-1^·FFM^-1^)	11.3(2.8)	11.3(1.9)	11.1(2.2)	22.8	0.6
VO_2 uptake_ (mL·min^-1^·kg^-1^)	6.9(2.1) ^a^	7.2(2.1) ^a^	19.3(3.1) ^b^	17.2	<0.0001
AOD (mL·min^-1^)	104.1(1.5) ^a^	102.1(1.5) ^a^	67.1(1.3) ^b^	21.9	<0.0001
Aerobic contribution (%)	6.4 ^a^	6.6 ^a^	22.5 ^b^	19.9	<0.0001
Anaerobic contribution (%)	93.6 ^a^	93.4 ^a^	77.5 ^b^	19.5	<0.0001

Values are expressed as the mean (standard deviation). *HR*: heart rate, *PO*: maximal power output developed during the supramaximal cycling exercise, *VO*_*2 uptake*_: oxygen uptake during repetition, *AOD*: accumulated oxygen, *%*: percentage.

Significant difference with OB (^a^: p<0.01). Significant difference from the 1^st^ repetition (^b^: p<0.01).

In this study the FFA levels were significantly associated to both the HOMA-IR index (r = 0.83, p<0.01) and aerobic contribution (r = 0.91, p<0.01) among adolescent boys.

## Discussion

To our knowledge, this study is the first to examine the effect of supramaximal exercise on plasma FFA concentrations in healthy adolescent boys across three body weight status groups. In the present study, the plasma FFA levels determined after SCE were significantly higher in the obese group than in normal-weight and overweight adolescents. Moreover, the relative anaerobic contributions to total energy release during bouts of SCE differed in obese adolescents compared with the other groups. Indeed, the anaerobic contributions to energy release decreased significantly in the OB group. These lower anaerobic contributions were accompanied by both lower AOD deficits and larger increases in oxygen uptake during SCE repetitions. These results suggest that in obese adolescent boys the energy supply seems to essentially consist of lipid mobilization and aerobic oxidation in response to SCE.

The performances demonstrated by participants during SCE satisfied the criteria for supramaximal testing. Indeed, the PO did not change between the sprints in the SCE in any of the three groups. The current knowledge on lipolytic activity in obese persons, especially during childhood, was primarily obtained from indirect calorimetry and indicates that there is reduced fat oxidation in response to acute submaximal and maximal exercise in obese persons [21]. In the present study, the plasma FFA concentrations measured at rest were significantly higher in obese boys than in normal-weight and overweight boys. The same results were also reported in adults showing that excess body fat is associated with an increased basal FFA concentration [22].

After the warm-up, the plasma FFA levels were similar to the rest values for normal-weight and overweight subjects but decreased significantly in obese boys and were lower than those of the normal-weight and overweight groups. To date, there is no information on the plasma FFA response to submaximal exercise in obese adolescents. Such information may be obtained in the present study if we consider the present warm-up (10 min at a submaximal power between 55–65 watts, corresponding to ~ 60% of VO_2max_) to be submaximal exercise. According to Pérez-Martin et al. [23], a lower lipid contribution to energy yield during a submaximal exercise test may be related to lower oxidative enzymatic activity in obese individuals. In adults, previous results showed a blunted lipolysis in obese women compared to normal-weight ones [24]. For these authors, the combination of heightened plasma insulin and diminished catecholamine and other counterregulatory hormone responses may account for subnormal plasma substrate increments in obese women during exercise. Additionally, Lanzi et al. [25] observed a blunted lipolysis in obese adults than in normal-weight during a submaximal incremental test reflecting the inhibitory role of increased insulin levels in lipolysis [26]. However, plasma FFA levels were higher probably due to greater adiposity and at high exercise intensities also due to the lower fat oxidation rate. This result suggests that the difference in the fat oxidation kinetics is likely linked to impaired muscular capacity to oxidize FFA in obese adults. Unfortunately, in the present work there is no information concerning the lipid oxidation that may be helpful to understand at least why plasma FFA level was lower than that reported in the adult study by Lanzi et al. [25].

The SCE was associated with a significant increase in the plasma FFA concentration only in the obese group. In normal-weight individuals, previous studies have shown that during intense exercise, there is a progressive decline in the plasma FFA response with increased blood glucose turnover responsible for energy yield [2]. However, the obese subjects exhibited higher plasma FFA levels than the normal-weight and overweight subjects. In fact, in both trained and untrained normal weight persons there is a reduction in fat oxidation in response to high intensity exercise that may be related to increased glycogen metabolism in muscle [27]. The limitation in fat use during high-intensity exercise stems in part from a decline in circulating fatty acids [28] during high-intensity exercise, beside of other contributing factors (e. g. muscle recruitment patterns, enzymatic capacities, substrate deliver) responsible of affecting substrate uptake during exercise [29]. Moreover, increasing FFA availability [30] as well as the differences in lipolysis between visceral and peripheral fat depots [31] constitute as well another factor affecting substrate uptake in obese individuals. In the present work, there is no data regarding the potential role of different fat depots on lipolysis during exercise.

Despite the fact that the quantity of plasma FFAs reflects the activity (increase vs. reduction) of lipolysis, the greater FFA concentrations suggest that plasma FFAs from hydrolysis of adipose tissue triglycerides can potentially meet the oxidative needs of working muscle [10]. In the present work, the determination of aerobic and anaerobic contributions suggest that OB adolescents rely more on the aerobic contribution (e.g., oxidative processes). Actually, it has been identified that the energy supply needed to perform a supramaximal event such as found in SCE results from anaerobic contributions [3, 4] as revealed by both the OW and NW groups. However, contrary to the above findings, the aerobic contributions to SCE performance in OB adolescent boys represent a significant portion of the energy supply which may result in lower rates of anaerobic glycolysis. This tendency, previously reported in normal-weight subjects [32], suggests that this incompatibility between anaerobic energy release and power output during supramaximal exercise is sometimes compensated for by the increased contributions of aerobic metabolism as reflected by the increase in oxygen consumption. Furthermore, the index of insulin resistance was deteriorated in obese adolescents. Indeed, it has been reported that insulin resistance diminished glucose transport and reduced glycogen synthase and pyruvate dehydrogenase activity [30]. These abnormalities account for disturbances in the two major intracellular pathways of glucose disposal, glycogen synthesis and glucose oxidation [33]. Consequently, the increase in aerobic contribution observed in the current work reflects an increase in oxygen consumption to oxidize substrate which may be derived from FFA metabolites to supply energy for working muscle. This hypothesis is reinforced by the fact that we found a significant positive correlation between aerobic contribution and plasma FFA levels in response to SCE.

However, there are some limits in the current study. Firstly, the missing post-exercise changes in several metabolic and hormonal parameters (ex. insulin) may allow us to better understand the effect of such exercise on metabolic adaptations. Secondly, the standardized breakfast was not directly controlled during the study, then we cannot be sure if our instructions were fully respected. Finally, our study participants were asked to rest the day before all testing. However, no one can assume that the participation to 1.5 hours physical education 36 to 48 hours before our experiment would not have any consequences on our blood measurements.

## Conclusion

The FFA concentrations measured immediately after SCE were higher in the OB group than in the OW and NW groups. Moreover, the aerobic contributions to SCE were significantly higher in obese adolescents and increased significantly during the 2^nd^, 3^rd^ and 4^th^ repetitions. Despite the general acceptance that higher basal levels of plasma FFAs is an indication of metabolic abnormality in obese individuals, the increase of this metabolite during SCE may reflect higher lipid solicitation to supply energy to working muscles. In addition, the higher aerobic contribution observed in obese adolescent boys compared to normal-weight and overweight groups via SCE may suggest the existence of an alternative approach to deliver energy by FFA oxidation.
